# Molecular Markers for Analyses of Genetic Diversity within the *Anastrepha fraterculus* Complex with Emphasis on Argentine Populations

**DOI:** 10.3390/insects15100748

**Published:** 2024-09-27

**Authors:** Ludvik M. Gomulski, María Teresa Vera, Silvia B. Lanzavecchia, Riccardo Piccinno, Giulia Fiorenza, Daniel De Luca, Beatriz N. Carrizo, Juan Pedro R. Bouvet, Valeria A. Viana, Carlos Cárceres, Walther Enkerlin, Anna R. Malacrida, Giuliano Gasperi

**Affiliations:** 1 Department of Biology and Biotechnology, University of Pavia, 27100 Pavia, Italy; ludvik.gomulski@unipv.it (L.M.G.); riccardo.piccinno@unipv.it (R.P.); giulia.fiorenza01@universitadipavia.it (G.F.); daniel.deluca@unipv.it (D.D.L.); annarodolfa.malacrida@unipv.it (A.R.M.); 2 Facultad de Agronomía, Zootecnia y Veterinaria, Universidad Nacional de Tucumán, Tucumán T4100, Argentina; teresa.vera@faz.unt.edu.ar; 3 Laboratorio de Insectos de Importancia Agronómica, Instituto de Genética Ewald A. Favret (INTA), Buenos Aires B1713, Argentina; lanzavecchia.silvia@inta.gob.ar; 4 Estación Experimental Agrícola Famaillá, Instituto Nacional de Tecnología Agropecuaria (INTA), Tucumán T4132, Argentina; carrizo.beatriz@inta.gob.ar; 5 Grupo de Protección Vegetal, EEA Concordia, Instituto Nacional de Tecnología Agropecuaria (INTA), Entre Ríos E3200, Argentina; bouvet.juan@inta.gob.ar (J.P.R.B.); viana.valeria@inta.gob.ar (V.A.V.); 6 Insect Pest Control Section, Joint FAO/IAEA Centre, 1400 Vienna, Austria; carceresc2@gmail.com (C.C.); w.r.enkerlin@iaea.org (W.E.)

**Keywords:** *Anastrepha fraterculus* complex, *Anastrepha schultzi*, ITS2, species complex, morphotypes, Argentina

## Abstract

**Simple Summary:**

The South American fruit fly *Anastrepha fraterculus* (Wiedmann) is found from northern Mexico to northern Argentina, where it causes damage to many different wild and cultivated fruits. It is a not a single species, but a complex of practically identical species. Eight morphological types (morphotypes) have been identified. To facilitate the identification of the species, alternative, non-morphological methods, such as those based on genome sequences, are necessary. The sterile insect technique is an efficient method used to combat these pests, which involves the release of many sterile male insects into the wild. Mass-reared sterile field-released males mate with females, which lay inviable eggs, thereby reducing the population. This approach is only successful when the released males are sexually compatible with the females of the target population. Hence, accurate identification is necessary for its success. We evaluated the use of the internal transcribed spacer 2 (ITS2) sequence for discriminating members of the *A. fraterculus* cryptic species complex and a related species, *Anastrepha schultzi* Blanchard. The ITS2 sequence successfully discriminated between different morphotypes and provides a basis for the development of keys for discrimination of the species within the complex. ITS2 also represents an important marker for the poorly studied species *A. schultzi*.

**Abstract:**

The South American fruit fly *Anastrepha fraterculus* (Wiedmann) has a vast range extending from northern Mexico, through Central America, to South America where it is an extremely polyphagous pest of wild and cultivated fruits. It is a complex of cryptic species currently composed of eight recognised morphotypes: “Mexican”, “Venezuelan”, “Andean”, “Peruvian”, “Ecuadorian”, and the three Brazilian morphotypes “Brazilian-1”, “Brazilian-2”, and “Brazilian-3”. Molecular markers that can identify the member species of the complex are crucial for the implementation of effective pest control measures, such as the sterile insect technique. The object of this study was to evaluate the use of the internal transcribed spacer 2 (ITS2) sequence for discriminating several members of the *A. fraterculus* cryptic species complex (Mexican, Peruvian, and Brazilian-1) and a related species, *Anastrepha schultzi* Blanchard. The analysis highlighted significant genetic differentiation between the evaluated morphotypes, allowed their discrimination within the *A. fraterculus* cryptic species complex, and provided new insights into their genetic relationships. The ITS2 marker provides a basis for the development of taxonomic keys for the discrimination of the cryptic taxa within the *A. fraterculus* cryptic species complex. ITS2 also represents an important marker for the poorly studied species *A. schultzi*.

## 1. Introduction

Accurate species identification is essential for effective pest eradication programmes. This can be especially difficult when rapid species radiation has produced several closely related cryptic species. Among the true fruit flies (Diptera: Tephritidae), and within the genus *Anastrepha*, the *fraterculus* species group includes at least 34 species [1,2]. One member of the *fraterculus* species group, *A. fraterculus* (Wiedemann), is commonly referred to as the South American fruit fly. This extremely polyphagous pest of cultivated fruits, has a vast range extending from northern Mexico to South America [3,4,5]. *Anastrepha fraterculus* has long been known to be a cryptic species complex, with Stone [6,7], in 1942, describing extensive morphological variation throughout its range. Numerous studies using a variety of characteristics including egg morphology, karyotype, isozymes, multivariate analysis, and mating compatibility have identified eight forms, morphotypes, or putative species in the cryptic species complex [3,8,9,10,11,12,13,14,15,16,17]. Three of the morphotypes are Brazilian: “Brazilian-1”, which also includes populations from Argentina, Paraguay, Boliva, and highland southeastern Peru, “Brazilian-2”, and “Brazilian-3”. The other morphotypes are “Mexican”, including populations from Mexico, Costa Rica, Colombia, Guatemala, and Panama; “Venezuelan”, from the lowlands of Venezuela and the Caribbean; “Andean”, from the highlands of Venezuela and Colombia; “Ecuadorian”, from the highlands of Ecuador and Peru; and “Peruvian”, from the lowlands of Peru and Ecuador and southern Colombia [8,18].

The identification of these distinct morphotypes within the range of *A. fraterculus* stimulated the search for correlated molecular markers. The development of such markers is crucial for identifying the target species, allowing effective pest control measures, such as the sterile insect technique (SIT), to be implemented. The SIT is an environmentally friendly strategy involving the inundation of an area with sterile male insects. These males mate with wild females, thereby reducing the population. This approach yields significant results when the released males belong to the same species or strain as the target population. However, it becomes ineffective if the sterile and target populations are not of the same species or if their mating behaviour is incompatible. Hence, accurate identification is pivotal for the success of this method.

Among the molecular markers previously studied was the mitochondrial cytochrome oxidase I gene (COI). This gene is well conserved at the intraspecific level, but often differs sufficiently between species to define interspecific boundaries [19]. Although COI has been shown to discriminate many species within the genus *Anastrepha*, it is unable to resolve many members of the *fraterculus* species group [20,21], and even less so members of the *A. fraterculus* cryptic species complex. A more promising molecular marker, the ribosomal internal transcribed spacer 1 (ITS1), was analysed by Sutton and colleagues from wild samples of the *A. fraterculus* cryptic species complex from Argentina, Bolivia, Peru, Ecuador, Colombia, Brazil, Venezuela, Guatemala, and Mexico [18]. The authors identified four non-randomly distributed ITS1 sequence groups, consistent with the known morphotypes [18]. Prezotto and colleagues [8] used a combined analysis of ITS1 and morphometric data to discriminate six members of the *A. fraterculus* cryptic species complex (Brazilian-1, Brazilian-2, Brazilian-3, Peruvian, Andean, and Mexican) and found that morphometric characterisation based on female wing shape was highly congruent with genetic data obtained through ITS1 analysis, which revealed specific sequences for each morphotype. Scally and colleagues [22] used nine loci (seven nuclear and two mitochondrial) and found incongruences between the nuclear and mitochondrial datasets, which they hypothesised was a result of introgression to which mitochondrial markers are more susceptible. Barr and colleagues [23], using a fragment of the ribosomal internal transcribed spacer 2 (ITS2) sequence, were able to distinguish *Anastrepha suspensa* (Loew) from other species of *Anastrepha*, including members of the *fraterculus* cryptic species complex, indicating the utility of the ITS2 marker for *Anastrepha* species identification. Selivon and colleagues [12] used an integrated analysis of multiple biological characteristics (mitotic chromosomes, ITS1, mating behaviour, and wing shape) on three morphotypes of the *A. fraterculus* cryptic species complex present in Brazil (Brazil-1, Brazil-2, and Brazil-3), and found morphotype-specific differences in the sex chromosomes and ITS1 sequences. The ITS1 sequence has also recently been successfully used in a phylogeographical analysis that considered individuals from 73 localities in 10 countries across the Americas [24].

The objective of this study is to evaluate the use of a larger fragment comprising part of the 5.8S ribosomal gene, the ITS2A sequence, the 2S sequence, the ITS2 sequence, and part of the 28S ribosomal gene for discriminating members of the *A. fraterculus* cryptic species complex. We also include samples of another member of the *fraterculus* species group, *A. schultzi* Blanchard. This species has, to date, been found only in Peru and Argentina, where it competes with *A. fraterculus* for resources [25]. *Anastrepha schultzi* can be distinguished from members of the *A. fraterculus* cryptic species complex using the morphology of the genitalia, but very limited information is available on this species. At the time of writing (July 2024), only 15 sequences are available for *A. schultzi* in GenBank. Our aim is to derive information on the genetic status of these flies and on their relationship with the *A. fraterculus* cryptic species complex.

## 2. Materials and Methods

### 2.1. Samples and DNA Extraction

The samples used in this study included five laboratory strains and twelve wild-collected samples. The laboratory strains, indicated by their capitalised names, were TEOCELO (derived from Teocelo, Veracruz State, Mexico), a representative of the Mexican morphotype; LA MOLINA (derived from La Molina, Lima Province, Perú), a representative of Peruvian morphotype 1; VACARIA (derived from Vacaria, Rio Grande do Sul State, Brazil) and HORCO MOLLE (derived from Horco Molle, San Miguel de Tucumán, Tucumán Province, Argentina), representatives of Brazilian morphotype 1; and PIRACICABA (derived from Piracicaba, Sao Paulo State, Brazil), considered to be a representative of Brazilian morphotype 1.

**Figure 1:**
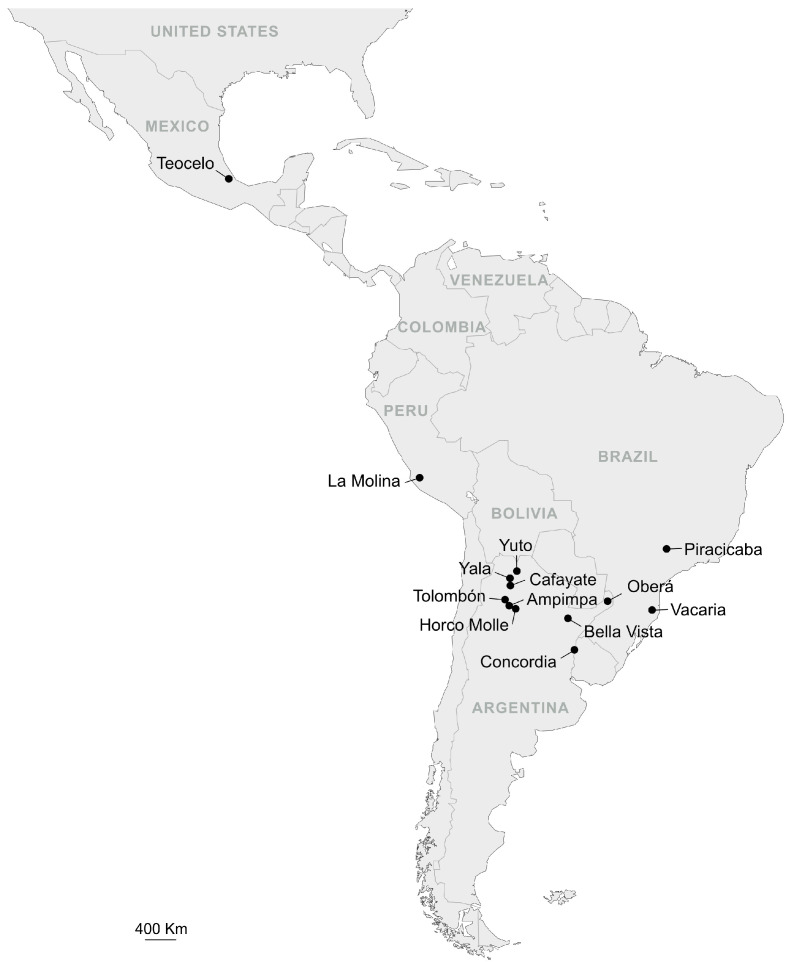
Geographic locations of the sites of collections that gave rise to the laboratory strains and of the wild samples considered in the study.

The wild-captured samples were Horco Molle-W and Horco Molle-P collected from tropical walnuts (*Juglans australis* Griseb) and peaches (*Prunus persica* (L.) Batsch), respectively, at Horco Molle, San Miguel de Tucumán, Tucumán Province, Argentina; Tolombón-Pr from pears (*Pyrus communis* L.), at Tolombón, Salta Province, Argentina; Cafayate-P from peaches at Cafayate, Salta Province, Argentina; Yala-W from tropical walnuts at Yala, Jujuy Province, Argentina; Yuto-G from guava (*Psidium guajava* L.) at Yuto, Jujuy Province, Argentina; Concordia-G from guava at Concordia, Entre Ríos Province, Argentina; Bella-Vista-G from guava at Bella-Vista, Corrientes Province, Argentina; Oberá-G from guava, at Oberá, Misiones Province, Argentina; and Ampimpa-P from peaches, Tucumán Province, Argentina. Two samples of another species, *Anastrepha schultzi*, were also considered: Horco-Molle-W-schultzi from tropical walnuts at Horco Molle, San Miguel de Tucumán, Tucumán Province, Argentina, and Yala-W-schultzi from tropical walnuts at Yala, Jujuy Province, Argentina (Figure 1; Table 1). All of the laboratory and wild samples were supplied as ethanol-preserved specimens from the International Atomic Energy Agency (IAEA), Seibersdorf, Austria. The laboratory colony samples were sent to the Department of Biology and Biotechnology, University of Pavia, Italy, in 2019.

DNA was extracted from whole male and female individuals of the laboratory strains. In the case of wild-caught specimens, DNA was extracted from two to three legs so that the morphological identification of the otherwise intact specimens, preserved individually in ethanol at the University of Pavia, could be re-evaluated if necessary. The alcohol-preserved whole specimens or legs were rinsed for 15 min in distilled water and then processed using the Baruffi method [26], and resuspended in TE (10 mM Tris–HCl, pH8, 1 mM EDTA). The DNA was quantified using a Nanodrop ND-1000 spectrophotometer (Nano-drop Technologies Inc., Wilmington, DE, USA) and then diluted in water to a concentration of ~5 ng/µL.

### 2.2. ITS2 Marker Characterisation

Sequences covering parts of the 5.8S, ITS2, and 28S nuclear ribosomal DNA region of *A. fraterculus* (GenBank accession numbers AY775552.1, AF210891.2, KT594240.1–KT594243.1, KT594245.1, and KT594002.1–KT594006.1; Garma & Haymer, unpublished; Sonvico et al. unpublished; [22]) were aligned to the corresponding nuclear ribosomal DNA region of *A. suspensa* (GenBank accession DQ279855.1; [27]) and the Mediterranean fruit fly, *Ceratitis capitata* (Wiedemann) (GenBank accessions AF189691.2 and KC177754.1; [28,29]) to identify the limits of the 5.8S ribosomal gene, the initial portion of the ITS2 sequence, and part of the 28S ribosomal gene in *A. fraterculus*. Specific PCR primers were designed in the identified 5.8S region (A.fra5.8Sf: 5′ CACATGAACATCGACATTTTGAAC 3′) and in the 28S region (A.fra28Sr TTTAATATAACTCAATGACTTGCACA) using Primer 3 [30] (Table S1).

PCR amplifications were performed using the AccuPrime Taq DNA Polymerase High Fidelity Kit (Life Technologies SrL, Monza, Italy) using the following cycle conditions: 94 °C for 2 min, 30 cycles at 94 °C for 30 s, 55 °C for 30 s, 72 °C for 1 min 40 s, and a final extension at 72 °C for 10 min using the A.fra5.8Sf and A.fra28Sr rDNA primers. Reactions were performed in a volume of 25 µL with approximately 5 ng DNA, 60 mM Tris-SO_4_ pH 8.9, 18 mM (NH_4_)_2_SO_4_, 2 mM MgSO_4_, 200 µM dNTP, 20 pmol of each primer, and 1 unit of AccuPrime Taq DNA polymerase. Amplification products were electrophoresed on 1.5% agarose gels together with a 100 bp ladder (Life Technologies SrL) and visualised using Ethidium bromide staining under UV illumination.

The amplification products were cloned using the TOPO TA cloning kit (Invitrogen, Monza, Italy) and both strands of multiple clones were sequenced using Sanger sequencing chemistry (Macrogen Europe, Amsterdam, Netherlands). The sequences were assembled using CLC Main Workbench 6.9.1 (CLC bio, Aarhus, Denmark) and the resulting sequences were again aligned with the corresponding nuclear ribosomal DNA region of *C. capitata* (AF189691.2 and KC177754.1; [28,29]) and *A. suspensa* (DQ279855.1; [27]). These alignments permitted the identification of the 5.8S/ITS2A/2S/ITS2/28S junction positions.

Having identified the limits of the ITS2 sequence, primers were designed to amplify a smaller amplification product containing 22 bp of 5.8S ribosomal gene, the ITS2A sequence, the 2S sequence, the ITS2 sequence, and 80 bp of the 28S ribosomal gene (excluding primer sequences). The primers chosen were the original 5.8S forward primer (A.fra5.8Sf: 5′ CACATGAACATCGACATTTTGAAC 3′) and a new reverse primer A.fraITS2r: 5′ TTTTCGCTCGCCGCTACTAA 3′ within the 28S gene (Table S1).

Using this new pair of primers, PCR amplifications were performed using Taq DNA polymerase (Life Technologies SrL) using the following cycle conditions: 94 °C for 2 min, 30 cycles at 94 °C for 30 s, 56 °C for 30 s, 72 °C for 1 min, and a final extension at 72 °C for 10 min. Reactions were performed in a volume of 25 µL with approximately 5 ng DNA, 20 mM Tris-HCl pH 8.4, 50 mM KCl, 1.5 mM MgCl_2_, 200 µM dNTP, 20 pmol of each primer, and 1 unit of Taq DNA polymerase. Amplification products were electrophoresed on 1.5% agarose gels together with a 100 bp ladder (Life Technologies SrL) and visualised using Ethidium bromide staining under UV illumination. Amplification products were cloned using the TOPO TA cloning kit (Invitrogen) and both strands of multiple clones were sequenced (Macrogen Europe).

### 2.3. Data Analyses: Variability and Differentiation

The sequences were aligned using MAFFT version 7 [31] using the Q-INS-i strategy. Using this alignment, the ITS2 nucleotide diversity and haplotype diversity [32] parameters were determined using the program ARLEQUIN 3.5 [33] based on pairwise difference distance matrix and significance-tested using 10,000 permutations (all remaining parameters given default values). An analysis of molecular variance (AMOVA) [33] was performed using ARLEQUIN 3.5 to hierarchically partition genetic diversity within populations and between populations, and within and among individuals within populations and between populations based on the pairwise difference distance matrix and significance-tested using 10,000 permutations (all remaining parameters were given default values).

Principal component analysis (PCA) was performed using the method of Konishi and colleagues [34]. Phylogenetic analyses of the sample ITS2 sequences were performed using Maximum Likelihood (IQ-TREE v. 2.3.4) [35] using the model determined within the ModelFinder option [36], and Bayesian methods (MrBayes 3.2.7a) [37] using the model identified by modeltest-ng v. 0.1.7 [38].

## 3. Results

### 3.1. Characterisation of the ITS2 Locus

**Figure 2:**
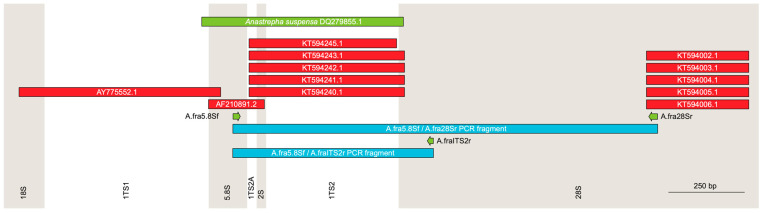
Scheme of the characterisation of the ribosomal DNA region in *Anastrepha fraterculus* using sequences of *A. fraterculus* (red) and *A. suspensa* (green) available in GenBank. The 18S, ITS1, 5.8S, ITS2A, 2S, ITS2, and 28S regions and the positions of the primers and the amplification products (blue) are shown.

The manual alignment of the available sequences in GenBank covering parts of the 5.8S, ITS2, and 28S nuclear ribosomal DNA region of *A. fraterculus* (AY775552.1, AF210891.2, KT594240.1–KT594243.1, KT594245.1, and KT594002.1–KT594006.1) resulted in two non-contiguous sequences of 1268 bp and 337 bp that included part of the 5.8S ribosomal gene, the initial portion of the ITS2 sequence, and part of the 28S ribosomal gene. These sequences were used to design the first set of specific primers (A.fra5.8Sf and A.fra28Sr) that amplified fragments from HORCO MOLLE and TEOCELO genomic DNA that once sequenced produced fragments of 1329–1335 bp (excluding primer sequences), consisting of part of 5.8S (22 bp), the ITS2A sequence (30 bp), the 2S sequence (30 bp), and the ITS2 sequence (435–436 bp in TEOCELO, and 440–441 bp in HORCO MOLLE) and 812 bp of 28S (Figure 2; the ten sequences, five from each strain, have been deposited in GenBank with accession numbers PQ111866-PQ111875).

These sequences permitted the design of a primer, A.fraITS2r, that, together with the original A.fra5.8Sf primer, amplified a smaller amplification product of about 641–647 bp that, after excluding the primer sequences, included 22 bp of 5.8S ribosomal gene, the ITS2A sequence, the 2S sequence, the ITS2 sequence, and 80 bp of the 28S ribosomal gene (Figure 2).

In total, 151 ITS2 sequences were obtained from 62 individual flies from the laboratory strains and from the wild population samples (Table 2). The sequences were named according to their origin, the sex of the individual, the individual number, and a letter indicating the clone, i.e., “Concordia M2c” indicates the third clone derived from a male individual no. 2 from the Concordia sample. The ITS2 sequences, complete with flanking regions (but excluding primers), ranged from 580 bp (TEOCELO) to 607 bp (Horco Molle-P). The sequences had a low content of GC nucleotides (20.04–20.54%), similar to that of the same region in *C. capitata* (23.9% GC, AF189691.2). The sequences have been deposited in GenBank with accession numbers PQ111876–PQ112026.

The MAFFT alignment of the 152 sequences (including the *A. suspensa* DQ279855.1 sequence) was 638 bp in length.

### 3.2. ITS2 Sequence Variability

The presence of sequence haplotypes was determined using ARLEQUIN 3.5. All of the samples analysed contained multiple ITS2 sequence haplotypes, ranging from 15 haplotypes from six individuals in Ampimpa-P, to two haplotypes from a single individual from Horco Molle-W. Within three of the samples, LA MOLINA, VACARIA, and *A. schultzi* Horco Molle-W, all sequences obtained represented different haplotypes. Taken together, the 151 sequences obtained from the 62 individuals analysed represented a total of 97 different sequence haplotypes (Table 2).

Estimates of haplotype diversity and nucleotide diversity derived from the sequences obtained from the laboratory and wild samples indicated that the samples were similar in their levels of variability. The haplotype diversity values were high, ranging from 0.70 in Tolombón to 1.00 in the LA MOLINA and VACARIA strains and the Horco Molle *A. schultzi* sample. The sequences derived from laboratory strain TEOCELO (Mexican morphotype) displayed a higher level of nucleotide diversity (π = 0.020) compared to the other laboratory strains (π = 0.005–0.008) and the wild samples (π = 0.001–0.008) (Table 2).

The molecular analysis of variance (AMOVA) including both the laboratory and wild samples of *A. fraterculus* indicated that there was a significantly greater proportion of variation among the samples (69.83%) than within the samples (30.17%) (Table 3). The *A. schultzi* samples were excluded from this analysis as they do not pertain to the *A. fraterculus* cryptic species complex.

When the AMOVA also considered variation within individuals, it was apparent that the contribution of the within-individual variation (21.25%) was greater than that of the within-sample variation (11.11%) (Table 4).

Grouping the samples with respect to their nominal morphotype classification, as indicated in Table 1, showed that most variation (75.58%) was found among morphotypes; a substantial amount of variation was present within the morphotypes (9.83%), whereas 14.58% was present within the samples (Table 5).

Finally, grouping the PIRACICABA haplotypes within the Peruvian morphotype, rather than with the Brazilian-1 morphotypes (see Sample Differentiation section, below) showed a much higher level of variation (84.67%) among morphotypes. Very little, though significant, variation was present within morphotypes (0.36%), whereas the remaining 14.97% variation was present within samples (Table 6).

### 3.3. Sample Differentiation

**Figure 3:**
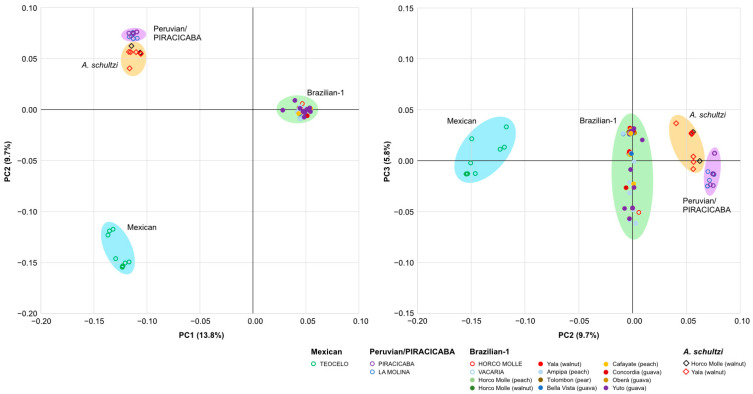
Principal component analysis on the ITS2 sequences from the different samples. PC1 vs. PC2 on the left and PC2 vs. PC3 on the right.

The results of the principal component analysis (Figure 3) clearly show the differentiation among the three *A. fraterculus* morphotypes and the *A. schultzi* samples. The samples belonging to the Brazilian-1 morphotype cluster together and are separated from the Mexican and Peruvian/PIRACICABA morphotype samples by the first axis (13.8%). The Mexican and Peruvian/PIRACICABA clusters are separated by the second axis (9.7%). The Peruvian/PIRACICABA and *A. schultzi* samples are distinct but closely associated, and are only partially separated by the third axis (5.8%).

ModelFinder within IQ-TREE v.2.3.4 identified the HKY+F [39] as the best-fit model and this was used to obtain the optimal Maximum Likelihood tree. A total of 100,000 ultrafast bootstrap replications [40] were performed with the -bnni option, which reduces the risk of overestimated bootstrap support values due to severe model violations. For the Bayesian analysis, Modeltest-ng v. 0.1.7 [38] identified the HKY model [39] as being the most appropriate model of sequence evolution, and this choice was confirmed in MrBayes by sampling sample across the entire general time reversible (GTR) model space in the Bayesian Markov chain Monte Carlo (MCMC) analysis [41].

**Figure 4:**
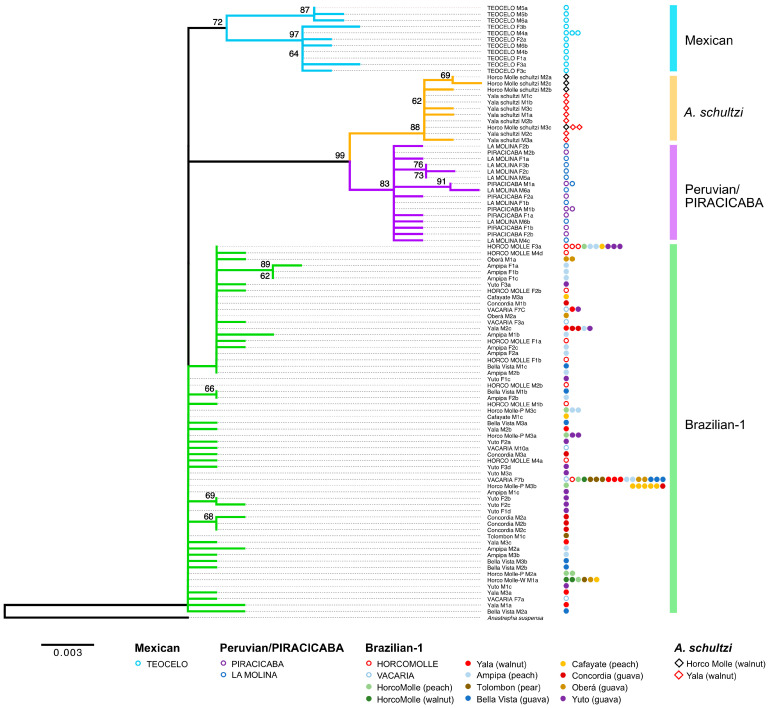
Maximum Likelihood tree of the ITS2 sequences from the different samples. Bootstrap numbers (percentage) of 100,000 replications are shown. The presence of haplotype sharing is indicated by strain/population-specific symbols.

**Figure 5:**
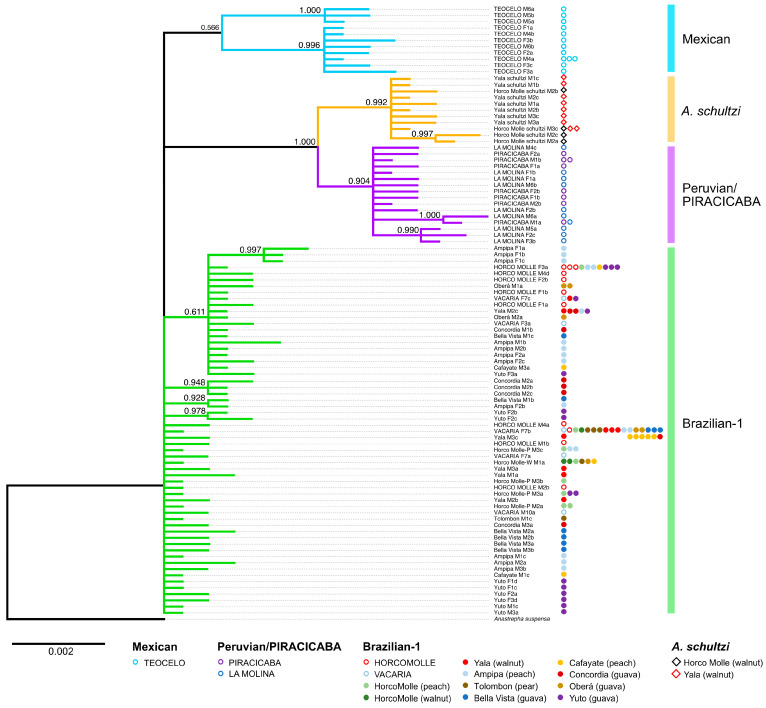
Bayesian analysis tree of the ITS2 sequences from the different samples. Values at the nodes are the posterior probabilities of each partition. The presence of haplotype sharing is indicated by strain/population-specific symbols.

Both the Maximum Likelihood and Bayesian trees (Figure 4 and Figure 5, respectively) share very similar topologies in displaying the relationships among the ITS2 haplotypes isolated from individuals belonging to the different samples of the *A. fraterculus* morphotypes and from *A. schultzi*.

In both trees with an *A. suspensa* outgroup, the Brazilian-1 haplotypes from the Argentine HORCO MOLLE strain from the wild Horco-Molle individuals from the walnut and peach hosts, from Yala, Ampipa, Bellavista, Cafayate, Concordia, Oberá, Yuto, and Tolombón, and the haplotypes from the Brazilian VACARIA strain do not form a cluster. The first cluster comprises the PIRACICABA strain haplotypes and the LA MOLINA sequences (Peruvian morphotype). The second cluster comprises the haplotype sequences from *A. schultzi*. Finally, the third cluster contains the haplotypes from the TEOCELO strain (Mexican morphotype).

In both the Bayesian and Maximum Likelihood trees, the three clusters are supported by moderately high to high posterior probability and bootstrap values, respectively. Several of the clusters are subdivided, for example, the Mexican cluster with subclusters supported by bootstrap values of 87 and 97, respectively. However, as haplotypes derived from the same individual fly fall in both clusters (TEOCELO M6a in one subcluster and TEOCELO M6b in the other), these subclusters cannot represent different taxonomic entities. In both trees, identical haplotypes were represented by a single representative sequence. Haplotype sharing is indicated by the presence of strain/population-specific symbols next to the name of the representative haplotype sequence name in Figure 4 and Figure 5.

## 4. Discussion

The findings of this study highlight significant genetic differentiation within the *A. fraterculus* cryptic species complex. The use of the ITS2 molecular marker allowed for detailed discrimination between different morphotypes and provided new insights into their genetic relationships. The ITS2 marker provides a basis for the development of taxonomic keys for the discrimination of at least some of the cryptic taxa within the *A. fraterculus* cryptic species complex. Furthermore, the *A. schultzi* samples appear to be related to those of the *A. fraterculus* Peruvian/PIRACICABA cluster.

### 4.1. Much Hidden Variation Is Present among and within the Morphotypes

The significant genetic variation among samples, as indicated by the molecular analysis of variance (AMOVA), highlights the underlying genetic architecture of the *A. fraterculus* cryptic species complex. A high degree of variability within the *A. fraterculus* cryptic species complex was also detected using a large set of microsatellite markers [42]. Grouping the samples with respect to their morphotype classification (AMOVA, Table 6) showed that most (almost 85%) of the variation was found among the morphotypes, less than 1% of the variation was present within the morphotypes, and the remaining 15% of the variation was present within the samples. The *A. schultzi* samples displayed a high degree of variability, with haplotype diversity estimates similar to those of their sympatric *A. fraterculus* counterparts in Horco Molle and Yala. This high level of variation in *A. fraterculus* and in *A. schultzi* underscores the importance of using accurate molecular markers for effective species identification and subsequent pest management strategies.

The principal component analysis (PCA) and phylogenetic analyses further supported the genetic differentiation among the morphotypes. The PCA clearly separated the Brazilian-1 morphotype from the Mexican and Peruvian/PIRACICABA morphotypes, while the Peruvian/PIRACICABA and *A. schultzi* samples were closely associated but distinct. Whether the genetic affinity of *A. schultzi* with the *A. fraterculus* Peruvian morphotype suggests a common ancestral origin is an open question. It is noteworthy, however, that *A. schultzi* was detected for the first time in Peru [43].

The phylogenetic trees, constructed using both Maximum Likelihood and Bayesian methods, displayed similar topologies and provided strong support for the clustering of haplotypes according to their respective morphotypes.

As in a recent study on members of the *A. fraterculus* cryptic species complex based on ITS1 sequences [24], we observed high levels of haplotype diversity and low levels of nucleotide diversity (π), which is indicative of small populations that have recently undergone expansion [44].

### 4.2. Genetic Differentiation and Morphotype Classification

One of the most surprising results was the clustering of the PIRACICABA strain haplotypes with those of the LA MOLINA Peruvian morphotype, rather than with haplotypes pertaining to the Brazilian-1 morphotype. Indeed, one haplotype represented by PIRACICABA M1a in both the ML and Bayesian trees is shared within both the LA MOLINA and PIRACICABA strains. Conventionally, the PIRACICABA strain has been classified as part of the Brazilian-1 morphotype. This differentiation of PIRACICABA with respect to the Brazilian-1 morphotype haplotypes is supported by earlier studies that reported prezygotic incompatibility between PIRACICABA and other Brazilian-1 populations from Vacaria and Tucumán [15,16], as well as differences in pheromone emission and morphometric characteristics [15,45]. These discrepancies question the classification of the PIRACICABA strain as pertaining to the Brazilian-1 morphotype and, instead, suggest a closer affinity to the Peruvian morphotype. It should also be noted that since both PIRACICABA and LA MOLINA are long-established laboratory strains, bottlenecks during the adaptation process may have favoured, in both cases, the prevalence of certain haplotypes. To better understand the relationships between PIRACICABA and LA MOLINA or other Brazilian-1 morphotype populations, new field collections are required.

This result, in conjunction with the *A. schultzi* clustering, provides new evidence of possible hybridisation events between ancestral populations (different morphotypes and/or closely related species) followed by a later dispersion and a recent population expansion [46,47]. These evolutionary events might be linked to anthropic activities affecting the host crops of fruit flies [47] involving ecological niche diversification and leading to local adaptation [24]. A more comprehensive sampling across Brazil should help resolve some discrepancies and identify unconclusive relationships among members of the *A. fraterculus* cryptic species complex and closely related species.

### 4.3. Implications for Pest Control

The ability to correctly differentiate between *A. fraterculus* morphotypes is crucial for developing targeted pest control measures. The sterile insect technique (SIT) is particularly dependent on accurate species identification, as misidentification can lead to ineffective control measures. Therefore, the development and application of molecular markers such as ITS2 can aid in the development of more effective and targeted control programmes, reducing the risk of incompatibility and enhancing pest control efficacy. This is particularly important as previously undetected morphotypes may be present in poorly characterised populations. Indeed, a recent phylogenomic analysis that included samples from uncharacterised populations identified a previously undescribed morphotype from Puerto Maldonado, Peru [46]. The Argentine populations analysed in this study were all confirmed to pertain to the same taxonomic entity, the Brazilian-1 morphotype. This is important supporting information for the efficient application of species-specific control programmes, such as the SIT, in Argentina.

## 5. Conclusions

This study highlights the utility of the ITS2 region as a molecular marker for delineating the genetic diversity within the *A. fraterculus* cryptic species complex. The findings underscore the need for ongoing genetic monitoring and the re-evaluation of morphotype classifications to inform pest management strategies. The ITS2 region also represents an important marker for studies of populations of *A. schultzi*. This is of particular relevance given that *A. schultzi* is a poorly known species that is closely related to the *A. fraterculus* cryptic species complex. The ITS2 sequence is one of many molecular markers that can be applied to species discrimination. The ITS2 region, being a relatively fast-evolving sequence, shows limited sequence variation within a species and, often, significant sequence divergence between closely related species [48]. This makes ITS2 valuable for inferring phylogenetic relationships across various taxa, including species complexes [23,49,50,51]. Despite the undoubted utility of the ITS2 region, the use of multiple molecular loci [22], or even entire genomes [46], together with other biological characteristics [12] should be explored as a means of increasing the resolutive power of such studies.

The scope of the current study was largely limited to Argentine populations of the *A. fraterculus* cryptic species complex. Future research should focus on further refining these, and other, genetic markers, and exploring their applicability to additional morphotypes across broader geographic ranges to improve our knowledge of the origin and adaptive radiation of these cryptic species.

## Figures and Tables

**Table 1:** Details of the *Anastrepha* samples collected in Argentina.

	Sample Name	Locality	Province	Coordinates	Host Fruit	Collection Year	Morphotype
*A. fraterculus*	Horco Molle-W	Horco Molle	Tucumán	26°48′10″ S 65°19′50″ W	Walnut	2019	Brazilian 1
	Horco Molle-P	Horco Molle	Tucumán	26°48′10″ S 65°19′50″ W	Peach	2019	Brazilian 1
	Ampimpa-P	Ampimpa	Tucumán	26°36′44″ S 65°50′31″ W	Peach	2021	Brazilian 1
	Tolombón-Pr	Tolombón	Salta	26°11′04″ S 65°56′25″ W	Pear	2019	Brazilian 1
	Cafayate-P	Cafayate	Salta	24°44′13″ S 65°23′21″ W	Peach	2019	Brazilian 1
	Yala-W	Yala	Jujuy	24°07′11″ S 65°24′17″ W	Walnut	2021	Brazilian 1
	Yuto-G	Yuto	Jujuy	23°35′01″ S 64°30′25″ W	Guava	2021	Brazilian 1
	Concordia-G	Concordia	Entre Ríos	31°22′37″ S 58°01′01″ W	Guava	2019	Brazilian 1
	Bella Vista-G	Bella Vista	Corrientes	28°30′46″ S 59°02′31″ W	Guava	2019	Brazilian 1
	Oberá-G	Oberá	Misiones	27°39′01″ S 55°24′10″ W	Guava	2019	Brazilian 1
*A. schultzi*	Horco Molle-W	Horco Molle	Tucumán	26°47′34″ S 65°18′58″ W	Walnut	2019	
	Yala-W	Yala	Jujuy	24°07′11″ S 65°24′17″ W	Walnut	2021	

**Table 2:** Features of the internal transcribed spacer 2 (ITS2) sequences in the different population samples.

Type	Sample	Morphotype	Individuals Analysed	Sequences Analysed	Haplotypes	Length (bp) ^1^	% GC	Haplotype Diversity ± SD	Nucleotide Diversity ± SD
*A. fraterculus*	TEOCELO	Mexican	6	13	11	580–598	20.54	0.962 ± 0.050	0.020 ± 0.011
(lab strains)	LA MOLINA	Peruvian	6	10	10	599–603	20.10	1.000 ± 0.045	0.008 ± 0.005
	VACARIA	Brazilian 1	3	5	5	601–604	20.12	1.000 ± 0.127	0.008 ± 0.006
	HORCO MOLLE	Brazilian 1	6	11	9	602–605	20.15	0.946 ± 0.066	0.006 ± 0.004
	PIRACICABA	Brazilian 1	4	8	7	599–603	20.08	0.964 ± 0.077	0.005 ± 0.003
*A. fraterculus*	Horco Molle-W	Brazilian 1	1	3	2	602–603	20.09	0.667 ± 0.314	0.001 ± 0.001
(wild samples)	Horco Molle-P	Brazilian 1	3	8	7	601–607	20.04	0.964 ± 0.077	0.007 ± 0.004
	Ampimpa-P	Brazilian 1	6	18	15	602–605	20.11	0.980 ± 0.024	0.007 ± 0.004
	Tolombón-Pr	Brazilian 1	2	5	3	599–603	20.10	0.700 ± 0.218	0.003 ± 0.003
	Cafayate-P	Brazilian 1	3	9	5	600–603	20.09	0.722 ± 0.159	0.004 ± 0.002
	Yala-W	Brazilian 1	3	9	7	601–606	20.13	0.917 ± 0.092	0.006 ± 0.004
	Yuto-G	Brazilian 1	6	16	13	600–605	20.12	0.967 ± 0.036	0.007 ± 0.004
	Concordia-G	Brazilian 1	3	8	7	600–603	20.07	0.964 ± 0.077	0.007 ± 0.004
	Bella Vista-G	Brazilian 1	3	9	7	601–604	20.14	0.917 ± 0.092	0.006 ± 0.004
	Oberá-G	Brazilian 1	2	6	4	602–603	20.14	0.867 ± 0.129	0.006 ± 0.004
*A. schultzi*	Horco Molle-W		2	4	4	596–600	20.52	1.000 ± 0.177	0.006 ± 0.005
(wild samples)	Yala-W		3	9	8	587–601	20.50	0.972 ± 0.064	0.008 ± 0.005
Total			62	151	97 ^2^				

^1^ Length excluding primer sequences. ^2^ Not the sum of the haplotypes due to haplotype sharing among samples. Haplotype diversity: equivalent to the expected heterozygosity for diploid data. It is defined as the probability that two randomly chosen haplotypes are different in the sample. Nucleotide diversity: the probability that two randomly chosen homologous nucleotide sites are different.

**Table 3:** Molecular analysis of variance (AMOVA) limited to between and within samples (excluding *A. schultzi*).

Source of Variation	d.f.	Sum of Squares	Variance Components	Percent of Variation	*p*
Among samples	14	702.459	5.274	69.83	<0.00001
Within samples	123	280.353	2.280	30.17	
Total	137	982.812	7.554		

**Table 4:** Molecular analysis of variance (AMOVA) between and within samples, and within individuals (excluding *A. schultzi*).

Source of Variation	d.f.	Sum of Squares	Variance Components	Percent of Variation	*p*
Among samples	14	702.459	5.107	67.63	<0.00001
Within samples	42	150.353	0.839	11.11	<0.00001
Within individuals	81	130.000	1.605	21.25	<0.00001
Total	137	982.812	7.552		

**Table 5:** Molecular analysis of variance (AMOVA) between and within the nominal morphotypes, and within samples (excluding *A. schultzi*).

Source of Variation	d.f.	Sum of Squares	Variance Components	Percent of Variation	*p*
Among morphotypes	2	514.798	11.814	75.58	<0.01271
Within morphotypes	12	187.660	1.537	9.83	<0.00001
Within samples	123	280.353	2.279	14.58	<0.00001
Total	137	982.812	15.229		

**Table 6:** Molecular analysis of variance (AMOVA) between and within morphotypes (grouping PIRACICABA within the Peruvian morphotype), and within samples (excluding *A. schultzi*).

Source of Variation	d.f.	Sum of Squares	Variance Components	Percent of Variation	*p*
Among morphotypes	2	669.375	12.895	84.67	<0.00001
Within morphotypes	12	33.084	0.055	0.36	0.01760
Within samples	123	280.353	2.279	14.97	<0.00001
Total	137	982.812	15.229		

## Data Availability

The sequence data presented in this study have been deposited in GenBank with accession numbers PQ111866-PQ112026.

## Supplementary Materials

The following supporting information can be downloaded at: https://www.mdpi.com/article/10.3390/insects15100748/s1, Table S1: Primer details.

## References

[1] Norrbom A.L., Zucchi R.A., Hernández-Ortiz V., Aluja M., Norrbom A. (1999). Phylogeny of the genera *Anastrepha* and *Toxotrypana* (Trypetinae: Toxotrypanini) based on morphology. Fruit Flies (Tephritidae): Phylogeny and Evolution of Behavior.

[2] Norrbom A.L., Muller A., Gangadin A., Sutton B.D., Rodriguez E.J., Savaris M., Lampert S., Clavijo P.A.R., Steck G.J., Moore M.R. (2021). New species and host plants of *Anastrepha* (Diptera: Tephritidae) primarily from Suriname and Par, Brazil. Zootaxa.

[3] Hernández-Ortiz V., Bartolucci A.F., Morales-Valles P., Frías D., Selivon D. (2012). Cryptic species of the *Anastrepha fraterculus* complex (Diptera: Tephritidae): A multivariate approach for the recognition of South American morphotypes. Ann. Entomol. Soc. Am..

[4] Steck G. (1991). Biochemical systematics and population genetic-structure of *Anastrepha fraterculus* and related species (Diptera, Tephritidae). Ann. Entomol. Soc. Am..

[5] Steck G.J. (1999). Taxonomic status of Anastrepha fraterculus In The South American Fruit Fly, Anastrepha fraterculus (Wied.): Advances in Artificial Rearing, Taxonomic Status and Biological Studies.

[6] Stone A. (1942). The fruit flies of the genus *Anastrepha*. U.S. Dep. Agric. Misc. Publ..

[7] Norrbom A.L. (2004). Host plant database for Anastrepha and Toxotrypana (Diptera: Tephritidae: Toxotrypanini). Diptera Data Dissemination Disk (CD-ROM) 2.

[8] Prezotto L.F., Perondini A.L.P., Hernández-Ortiz V., Frías D., Selivon D. (2019). What can integrated analysis of morphological and genetic data still reveal about the *Anastrepha fraterculus* (Diptera: Tephritidae) cryptic species complex?. Insects.

[9] Selivon D., Perondini A.L.P. (1998). Eggshell morphology in two cryptic species of the *Anastrepha fraterculus* complex (Diptera: Tephritidae). Ann. Entomol. Soc. Am..

[10] Selivon D., Vretos C., Fontes L., Perondini A.L.P., Barnes B.N. (2004). New variant forms in the *Anastrepha fraterculus* complex. Proceedings of the 6th International Symposium on Fruit Flies of Economic Importance.

[11] Selivon D., Perondini A.L.P., Morgante J.S. (2005). A genetic-morphological characterization of two cryptic species of the *Anastrepha fraterculus* complex (Diptera: Tephritidae). Ann. Entomol. Soc. Am..

[12] Selivon D., Perondini A.L.P., Hernandez-Ortiz V., doVal F.C., Camacho A., Gomes F.R., Prezotto L.F. (2022). Genetical, morphological, behavioral, and ecological traits support the existence of three Brazilian species of the *Anastrepha fraterculus* complex of cryptic species. Front. Ecol. Evol..

[13] Rull J., Abraham S., Kovaleski A., Segura D.F., Mendoza M., Liendo M.C., Vera M.T. (2013). Evolution of pre-zygotic and post-zygotic barriers to gene flow among three cryptic species within the *Anastrepha fraterculus* complex. Entomol. Exp. Et Appl..

[14] Hendrichs J., Vera M.T., De Meyer M., Clarke A.R. (2015). Resolving cryptic species complexes of major tephritid pests. Zookeys.

[15] Dias V.S., Silva J.G., Lima K.M., Petitinga C.S.C.D., Hernández-Ortiz V., Laumann R.A., Paranhos B.J., Uramoto K., Zucchi R.A., Joachim-Bravo I.S. (2016). An integrative multidisciplinary approach to understanding cryptic divergence in Brazilian species of the *Anastrepha fraterculus* complex (Diptera: Tephritidae). Biol. J. Linn. Soc..

[16] Vera M.T., Cáceres C., Wornoayporn V., Islam A., Robinson A.S., De La Vega M.H., Hendrichs J., Cayol J.P. (2006). Mating incompatibility among populations of the South American fruit fly *Anastrepha fraterculus* (Diptera: Tephritidae). Ann. Entomol. Soc. Am..

[17] Devescovi F., Abraham S., Roriz A.K.P., Nolazco N., Castañeda R., Tadeo E., Cáceres C., Segura D.F., Vera M.T., Joachim-Bravo I. (2014). Ongoing speciation within the *Anastrepha fraterculus* cryptic species complex: The case of the Andean morphotype. Entomol. Exp. Appl..

[18] Sutton B.D., Steck G.J., Norrbom A.L., Rodriguez E.J., Srivastava P., Alvarado N.N., Colque F., Landa E.Y., Sánchez J.J., Quisberth E. (2015). Nuclear ribosomal internal transcribed spacer 1 (ITS1) variation in the *Anastrepha fraterculus* cryptic species complex (Diptera, Tephritidae) of the Andean region. Zookeys.

[19] Hebert P.D., Cywinska A., Ball S.L., deWaard J.R. (2003). Biological identifications through DNA barcodes. Proc. Biol. Sci..

[20] Barr N.B., Ruiz-Arce R., Farris R.E., Silva J.G., Lima K.M., Dutra V.S., Ronchi-Teles B., Kerr P.H., Norrbom A.L., Nolazco N. (2018). Identifying *Anastrepha* (Diptera; Tephritidae) species using DNA barcodes. J. Econ. Entomol..

[21] Bartolini I., Rivera J., Nolazco N., Olórtegui A. (2020). Towards the implementation of a DNA barcode library for the identification of Peruvian species of *Anastrepha* (Diptera: Tephritidae). PLoS ONE.

[22] Scally M., Into F., Thomas D.B., Ruiz-Arce R., Barr N.B., Schuenzel E.L. (2016). Resolution of inter and intra-species relationships of the West Indian fruit fly *Anastrepha obliqua*. Mol. Phylogenet. Evol..

[23] Barr N., Ruiz-Arce R., Obregón O., Shatters R., Norrbom A.L., Nolazco N., Thomas D. (2017). Diagnostic characters within ITS2 DNA support molecular identification of *Anastrepha suspensa* (Diptera: Tephritidae). Fla. Entomol..

[24] Freilij D., Vilardi J.C., Gómez-Cendra P. (2024). Rapid cryptic divergence of the *Anastrepha fraterculus* complex in the Late Pleistocene: A phylogeographical-ecological approach. Biol. J. Linn. Soc..

[25] Blanchard E.E. (1961). Especies Argentinas del género *Anastrepha* Schiner (sens. lat.) (Diptera: Tephritidae). Rev. Invest. Agric. Buenos Aires.

[26] Baruffi L., Damiani G., Guglielmino C.R., Bandi C., Malacrida A.R., Gasperi G. (1995). Polymorphism within and between populations of *Ceratitis capitata*: Comparison between RAPD and multilocus enzyme electrophoresis data. Heredity.

[27] Fritz A.H. (2006). Sequence analysis of nuclear rDNA of *Anastrepha suspensa*. Ann. Entomol. Soc. Am..

[28] Douglas L.J., Haymer D.S. (2001). Ribosomal ITS1 polymorphisms in *Ceratitis capitata* and *Ceratitis rosa* (Diptera: Tephritidae). Ann. Entomol. Soc. Am..

[29] Wiegmann B.M., Trautwein M.D., Winkler I.S., Barr N.B., Kim J.W., Lambkin C., Bertone M.A., Cassel B.K., Bayless K.M., Heimberg A.M. (2011). Episodic radiations in the fly tree of life. Proc. Natl. Acad. Sci. USA.

[30] Untergasser A., Cutcutache I., Koressaar T., Ye J., Faircloth B.C., Remm M., Rozen S.G. (2012). Primer3—New capabilities and interfaces. Nucleic Acids Res..

[31] Katoh K., Rozewicki J., Yamada K.D. (2019). MAFFT online service: Multiple sequence alignment, interactive sequence choice and visualization. Brief. Bioinform..

[32] Nei M. (1987). Molecular Evolutionary Genetics.

[33] Excoffier L., Lischer H.E. (2010). Arlequin suite ver 3.5: A new series of programs to perform population genetics analyses under Linux and Windows. Mol. Ecol. Resour..

[34] Konishi T., Matsukuma S., Fuji H., Nakamura D., Satou N., Okano K. (2019). Principal Component Analysis applied directly to Sequence Matrix. Sci. Rep..

[35] Minh B.Q., Schmidt H.A., Chernomor O., Schrempf D., Woodhams M.D., von Haeseler A., Lanfear R. (2020). IQ-TREE 2: New models and efficient methods for phylogenetic inference in the genomic era. Mol. Biol. Evol..

[36] Kalyaanamoorthy S., Minh B.Q., Wong T.K.F., von Haeseler A., Jermiin L.S. (2017). ModelFinder: Fast model selection for accurate phylogenetic estimates. Nat. Methods.

[37] Ronquist F., Teslenko M., van der Mark P., Ayres D.L., Darling A., Höhna S., Larget B., Liu L., Suchard M.A., Huelsenbeck J.P. (2012). MrBayes 3.2: Efficient Bayesian phylogenetic inference and model choice across a large model space. Syst. Biol..

[38] Darriba D., Posada D., Kozlov A.M., Stamatakis A., Morel B., Flouri T. (2020). ModelTest-NG: A new and scalable tool for the selection of DNA and protein evolutionary models. Mol. Biol. Evol..

[39] Hasegawa M., Kishino H., Yano T. (1985). Dating of the human-ape splitting by a molecular clock of mitochondrial DNA. J. Mol. Evol..

[40] Hoang D.T., Chernomor O., von Haeseler A., Minh B.Q., Vinh L.S. (2018). UFBoot2: Improving the ultrafast bootstrap approximation. Mol. Biol. Evol..

[41] Huelsenbeck J.P., Larget B., Alfaro M.E. (2004). Bayesian phylogenetic model selection using reversible jump Markov chain Monte Carlo. Mol. Biol. Evol..

[42] Manni M., Lima K.M., Guglielmino C.R., Lanzavecchia S.B., Juri M., Vera T., Cladera J., Scolari F., Gomulski L., Bonizzoni M. (2015). Relevant genetic differentiation among Brazilian populations of *Anastrepha fraterculus* (Diptera, Tephritidae). Zookeys.

[43] Schliserman P., Ovruski S., Colin C., Norrbom A.L., Aluja M. (2004). First report of *Juglans australis* (Juglandaceae) as a natural host plant for *Anastrepha schultzi* (Diptera: Tephritidae) with notes on probable parasitism by *Doryctobracon areolatus*, *D-Brasiliensis*, *Opius bellus* (Braconidae) and *Aganaspis pelleranoi* (Figitidae). Fla. Entomol..

[44] Rosetti N., Remis M.I. (2012). Spatial genetic structure and mitochondrial DNA phylogeography of Argentinean populations of the grasshopper *Dichroplus elongatus*. PLoS ONE.

[45] Roriz A.K.P., Japyassú H.F., Cáceres C., Vera M.T., Joachim-Bravo I.S. (2019). Pheromone emission patterns and courtship sequences across distinct populations within *Anastrepha fraterculus* (Diptera-Tephritidae) cryptic species complex. Bull. Entomol. Res..

[46] Congrains C., Dupuis J.R., Rodriguez E.J., Norrbom A.L., Steck G., Sutton B., Nolazco N., de Brito R.A., Geib S.M. (2023). Phylogenomic analysis provides diagnostic tools for the identification of *Anastrepha fraterculus* (Diptera: Tephritidae) species complex. Evol. Appl..

[47] Diaz F., Luís A., Lima A., Nakamura A.M., Fernandes F., Sobrinho J.I., De Brito R.A. (2018). Evidence for introgression among three species of the *Anastrepha fraterculus* group, a radiating species complex of fruit flies. Front. Genet..

[48] Paredes-Esquivel C.C., Townson H. (2014). Functional constraints and evolutionary dynamics of the repeats in the rDNA internal transcribed spacer 2 of members of the *Anopheles barbirostris* group. Parasit Vectors.

[49] Wilkerson R.C., Reinert J.F., Li C. (2004). Ribosomal DNA ITS2 sequences differentiate six species in the *Anopheles crucians* complex (Diptera: Culicidae). J. Med. Entomol..

[50] Gomulski L., Meiswinkel R., Delécolle J., Goffredo M., Gasperi G. (2005). Phylogenetic relationships of the subgenus *Avaritia* Fox, 1955 including *Culicoides obsoletus* (Diptera, Ceratopogonidae) in Italy based on internal transcribed spacer 2 ribosomal DNA sequences. Syst. Entomol..

[51] Boykin L., Schutze M., Krosch M., Chomic A., Chapman T., Englezou A., Armstrong K., Clarke A., Hailstones D., Cameron S. (2014). Multi-gene phylogenetic analysis of south-east Asian pest members of the *Bactrocera dorsalis* species complex (Diptera: Tephritidae) does not support current taxonomy. J. Appl. Entomol..

