# Supplementary material for: Molecular Markers for Analyses of Genetic Diversity within the Anastrepha fraterculus Complex with Emphasis on Argentine Populations
Source: Insects. 2024 Sep 27;15(10):748. doi: 10.3390/insects15100748 (PMC11508799; doi:10.3390/insects15100748)
Supplement: Supplementary file 1 [file insects-15-00748-s001.zip › insects-3197699-supplementary.pdf]

Table S1. Primer details

Primer	Sequence (5′ – 3′)	Annealing temperature	Product size[1]	Application
A.fra5.8Sf	CACATGAACATCGACATTTTGAAC	55°C	1379 – 1385 bp	Determination of 5′ end of 28S gene
A.fra28Sr	TTTAATATAACTCAATGACTTGCACA	55°C		
A.fra5.8Sf	CACATGAACATCGACATTTTGAAC	56°C	624 – 651 bp	Amplification of region between
A.fraITS2r	TTTTCGCTCGCCGCTACTAA	56°C		the 5.8S and 28S genes

[1]including primers
